# National Gay Men’s HIV/AIDS Awareness Day — September 27, 2012

**Published:** 2013-09-20

National Gay Men’s HIV/AIDS Awareness Day is observed each year on September 27 to focus on the continuing effects of human immunodeficiency virus (HIV) infection and acquired immune deficiency syndrome (AIDS) on gay, bisexual, and other men who have sex with men (MSM) in the United States. By the end of 2009, an estimated 652,300 MSM, including 60,200 who were also injection drug users, were living with HIV infection, comprising 57% of persons living with HIV infection in the United States (1). MSM represent approximately 2% of the U.S. population (2); however, in 2010, MSM and MSM who were injection drug users accounted for 66% of all new HIV infections (3).

CDC supports a range of efforts to reduce HIV infection among MSM. These include HIV prevention services that reduce the risk for acquiring and transmitting HIV, increase diagnosis of HIV infection, and support the linkage of HIV-infected MSM to treatment and care. Additional information about CDC efforts to promote the health of MSM is available at http://www.cdc.gov/hiv/risk/gender/msm. Additional information about National Gay Men’s HIV/AIDS Awareness Day is available at http://www.cdc.gov/features/ngmhaad.
